# Nature and incidence of upper limb injuries in professional cricket players *a prospective observation*

**DOI:** 10.1186/1758-2555-4-42

**Published:** 2012-11-08

**Authors:** Mandeep S Dhillon, Bhavuk Garg, Ritesh K Soni, Himmat Dhillon, Sharad Prabhakar

**Affiliations:** 1 Department of Orthopaedics, Postgraduate Institute of Medical Education and Research, Chandigarh, Sec-12, Chandigarh, 160012, India

**Keywords:** Cricket, Injury, Upper limb, Bowler, Batsman

## Abstract

**Introduction:**

Cricket is the most popular sport in India, and is gaining in importance in all south-east Asian countries. The purpose of this study was to prospectively investigate the incidence, nature, and site of acute upper limb injuries sustained by professional cricketers of north India over a period of one year.

**Material & methods:**

95 cricket players (mean age 18.9 years) were prospectively evaluated for nature and incidence of upper limb injuries from 1st November 2008 to 31st October 2009. For the purpose of comparison the calculated injury incidence included injuries sustained during match as well as practice. Injuries were also grouped according to the type of cricket activities such as batting or fielding.

**Results:**

Out of 95 players evaluated, 24 were bowlers, 19 were batsmen, 8 were wicket keepers and the other 44 cricketers declared themselves as all rounders. There were a total of 16 upper limb injuries in 16 (16.8%) players. The majority of injuries (10/16) occurred while fielding. Out of 16 injuries, 11 were seen in hand, 3 were observed in elbow, while 2 patients suffered from shoulder problem. Twelve were acute injuries while 4 were classified as repetitive stress injuries (RSI).

**Conclusion:**

The incidence of upper limb injuries in cricketers at the professional and semi-professional level is significant, causing them to miss matches or practice for a significant number of days. This is the first study of Indian cricketers which documents the high incidence of upper limb injuries. The study highlights the importance of injury surveillance for Indian cricket. It is a concern which needs to be addressed by the players, coaches, teachers, administrators and medical personnel involved with cricket.

## Introduction

Cricket is the most popular sport in India and is gaining in importance in all South east Asian countries. Its expansion over the past decades has placed greater demands on cricketers due to increased playing hours and increased performance expectations [1]. However, despite being the most popular team sport in this region, there is not a single publication in the medical literature reporting cricket injuries from India or the subcontinent. Many of the previously published epidemiological data on cricket injuries, particularly at the elite level, have emanated from England, South Africa and Australia [2-5].

The incidence of cricket injuries has been reported in very few studies. A survey conducted by the British Sports Council [5] reported 2.6 injuries per 10,000 hours played. A similar survey conducted by the ACB [2] (Australian Cricket Board) reported the figure to be 24.2 per 10,000 player hours, which is considerably higher than the figures reported by the British survey. Injuries to the upper limb account for between 25% and 32% of all injuries. The most common types of injury are fractures, dislocations and contusions sustained during batting and fielding, especially finger injuries [6-9]. Shoulder injuries have also been reported, particularly in fielders [6,9].

Excluding the two previously cited surveys, the remaining literature pertaining to cricket injuries usually takes the form of isolated cases or case series with specific data on the incidence and nature of injuries incurred by professional cricketers is therefore limited. The purpose of this study was to prospectively investigate the incidence, nature, and site of acute upper limb injuries sustained by professional cricketers of north India over a period of one year.

## Material and methods

This was a prospective study to study the pattern of injuries in cricket players of North India. The study involved 95 players (composed of the Punjab Ranji Trophy team, the district teams of Punjab involved in cricket camps and the under 19 teams of the Punjab Cricket Association). The study period extended from 1st November 2008 to 31st October 2009 over one year comprising one playing season and one off season of six months each. The season in our set up is defined as time period when players play competitive matches whereas off season is defined as the one when players keep on practicing daily and often play local matches on weekends.

All the players associated with these teams were identified with the help of the Coaches and Physiotherapists and Trainers of the Punjab Cricket Association (PCA). They were evaluated at the start of the season, using the proforma designed at our institution. The player type was identified, and was categorized into a group i.e. bowler, batsman, wicket keeper and all-rounder. Categorization of the players was done on the basis of the description by the coaches and by the players themselves. Bowlers were further divided into fast and slow bowlers. A fast bowler [10] was defined as a bowler for whom the wicketkeeper would normally stand back from the stumps, due to the increased speed of the ball when bowled. All rounders were categorised as the ones who put equal effort both in bowling and batting during practice. The players who did not bowl, and were categorized as batsman.

These players were followed up regularly during the periodic training camps of PCA. The players were also serially contacted by telephone once every three months over one calendar year. The practice profile of the player, any history of injury (including number of days of practice/ number of matches missed) was recorded. The injuries were commonly reported by the physiotherapists and sometimes the researchers were directly informed of the injury by the players.

A detailed record of the practice profile of each player was kept. All injury definitions were as per the Cricket Australia model defined by Dr. John Orchard [11] with a few modifications. One unique definition included in the study has been the introduction of the term “all rounder” for purposes of player type classification. Most of the all rounders are batsmen who can bowl a bit, mostly slow spin bowling; some bowlers on the other hand exhibit more than average batting skills. Thus it was important to evaluate if this subgroup had a different injury or practice profile.

All injuries to the upper limb region were analyzed. The information obtained was recorded and entered in a computerized data base. Microsoft Excel (Redland, WA, U.S.A.) was used to store and analyze the data. For the purpose of comparison, the calculated injury incidence included injuries sustained during match as well as during practice.

Orchard et al [11] defined injury prevalence as the percentage of players missing through injury for each match. It was calculated by using a numerator of “missed player games” with a denominator of number of games, multiplied by number of squad members. However for this study, the prevalence was calculated based on the total number of “playing days” missed by the Cricketers due to injury. Hence the numerator used was missed player days multiplied by number of injured players with the denominator of average number of practice days multiplied by total number of players. To calculate this, the total number of days a player is either unable to play matches or practice cricket were taken into account. This is different from calculations based on only “match days” lost. So another deviation in this study from the international definitions was the inclusion of injuries which only caused missed time from practice.

## Results

Out of 95 players evaluated 24 were bowlers, 19 were batsman, 8 were wicket keepers and the other 44 cricketers declared themselves as all rounders. Among 24 bowlers 19 were fast bowlers and 5 were slow bowlers. Among 44 all rounders, 37 were spin bowlers and 7 were fast bowlers. Most of the all Rounders were batsmen with ability to spin the ball, or spinners with some batting ability. The average age of players in our study was 18.9 years (range 14–34 years) and average age of an injured player was 20.24years (range 15–30 years). The average number of years, player under study were playing professional cricket was 6.8 (range 1–15 years).

Apart from playing matches all the players practiced daily in PCA stadium Mohali and other stadiums of Punjab. We noted that the training sessions have a uniform time table throughout Punjab, which comprised 2 to 2.5 hours of physical training in the morning followed by 4 hours of game practice in the evening session. Practice is usually undertaken on six working days with a holiday on Sunday. However we found that most of the players, especially under 19 years, even played Local Street or club matches on Sundays (Table 1).

**Table 1:** Average practice profile of a player

	
No. of hours of daily practice	5.5± 0.35
No. of days of practice in a week	5.9±0.41
No. of weeks of practice in a year	41.7± 1.9
No. of days of practice in a year	245.4± 3.1
No. of hrs of practice in a year	1351.9± 6.8

There were a total of 16 upper limb injuries in 16 (16.8%) players (Table 2 &3). The majority of injuries (10/16) occurred while fielding. Four injuries were seen while bowling (2 fast bowlers, 1 in slow bowler, 1 all rounder), while 2 injuries occurred while batting. Out of 16 injuries, 11 were observed in hand, 3 were in elbow, while 2 patients suffered from shoulder problem. Out of 16 injuries, 12 were acute injuries while 4 were classified as repetitive stress injuries (RSI).

**Table 2:** Distribution of injuries according to type of player

	**BOWLING**	**FIELDING**	**BATTING**	**WICKETKEEPING**	**Total injuries**
ALL ROUNDERS	1	8	1	--	10
BATSMAN	---	--	1	--	01
BOWLER	3 (2 fast bowler, one slow bowler	2 (Both fast bowler)	--	--	05
WICKETKEEPER	---	---	--	--	
					16

**Table 3:** Distribution & Nature of Upper Limb Injuries in 95 players evaluated

**S.No.**	**Age (yrs)**	**Injury**	**Player**	**Activity during which injury sustained**	**Nature**	**Number of days practice missed**
**1.**	19	Mallet finger	All Rounder	Fielding	Acute	30
**2.**	22	Jersy finger	All Rounder	Fielding	Acute	40
**3.**	20	Mallet finger	(Fast bowler)	Fielding	Acute	32
**4.**	18	Gamekeeper thumb	All Rounder	Fielding	Acute	30
**5.**	16	Middle phalanx fracture	All Rounder	Fielding	Acute	50
**6.**	16	Gamekeeper thumb	Batsman	Batting	Acute	34
**7.**	22	Mallet finger	All Rounder	Fielding	Acute	29
**8.**	18	Metacarpophalangeal joint dislocation	(Fast bowler)	Fielding	Acute	41
**9.**	15	Bennett’s fracture	All Rounder	Fielding	Acute	40
**10.**	21	Scaphoid fracture	All Rounder	Fielding	Acute	43
**11.**	19	DeQuervain’s tenosynovitis	All Rounder	Bowling	Chronic RSI	21
**12.**	22	Lateral epicondylitis	All Rounder	Batting	Chronic RSI	23
**13.**	20	Medial epicondylitis	(Slow bowler)	Bowling	Chronic RSI	24
**14.**	19	Olecranon fracture	All Rounder	Fielding	Acute	60
**15.**	21	Acute biceps muscle strain	(Fast bowler)	Bowling	Acute	15
**16.**	26	Supraspinatus tendinitis	(Fast bowler)	Bowling	Chronic RSI	21

The average number of days a player missed practice / matches and was out of active cricket due to injury was 33.31 days, almost more than 1 month. The upper limb injury incidence per 10,000 hrs of practice for this study was 1.24. The upper limb injury incidence per 100 days of exposure was 0.07%.

## Discussion

The principal finding of this study is that upper limb injuries are common in cricket players. The fingers were the most common site of injury. A similar observation has been made by other studies as well [6-9]. Specific studies on the incidence and nature of upper-limb injuries incurred by cricketers are limited. In this study, most of the upper limb injuries were sustained during fielding; all rounders were the most often affected. Finger injuries usually occurred as a result of being struck by the ball during fielding, catching and batting. Out of the total injuries, 62.5% of injuries were severe enough to prevent the cricketer from returning to play for more than 4 weeks. On comparing the incidence of injury in All Rounders and other players, there was marked increased susceptibility to upper-limb injuries in all rounders (Table 2 &3).

Many previous studies [2,9], especially those from Australia, have focused on players during the playing season or during matches; leading to calculations based on *“match hours”* lost, and incidence and prevalence rates have been calculated on this basis. Some aspects of playing cricket are unique to India, we found that most of our players continued to practicing/playing Cricket during the so called *“off season”* (summer months) also, and so the total playing time becomes much more in one calendar year. For this reason our study was designed to extend for one full year (i.e. 1st November 2008 to 31st October 09). This method of reporting has not been documented previously in any similar study on cricket players.

The Upper limb injury incidence in cricketers studied was calculated to be 1.24 per 10,000 hours of play. This has been found to be relatively low incidence when compared with other studies [2-7]. Upon analyzing their data, it was observed that they have calculated their injury incidence based on the injuries sustained during matches and involved an elite international level cohort. Ours was a different methodology, as our calculations were based mainly upon injuries sustained during practice and involved a larger number of under 19 and under 17 players of the PCA and total number of playing hours also included time spent in physical training. In our opinion, international players anyway play more intense matches, and injury rates could be higher.

The incidence of upper limb injuries in cricketers at the professional and semi-professional level is significant. Consequently the players miss matches or practice for a significant number of days. This is the first study examining Indian cricketers and the high incidence of upper limb injuries found in this research should be addressed by the players, coaches, teachers, administrators and medical personnel involved with cricket. All rounders, especially while fielding, were found to be most susceptible to upper limb injuries. Injury surveillance in cricket in India is still in its infancy. It is under-funded, its importance unrecognized and its potential to improve Indian cricket remains unrecognized. Publication of such data has the potential to stimulate more funding and research efforts towards injury surveillance and pre participation evaluation of cricketers in the whole of the subcontinent.

## Competing interests

The authors declare that they have no competing interests.

## Authors’ contribution

MSD is the principal investigator and treated all the cricketers, BG and SP prepared the manuscript, HD was the physiotherapist as well as collected data along with RKS. All authors read and approved the final manuscript.
